# ^1^H-NMR Profiling and Chemometric Analysis of Selected Honeys from South Africa, Zambia, and Slovakia

**DOI:** 10.3390/molecules23030578

**Published:** 2018-03-05

**Authors:** Emmanuel O. Olawode, Roman Tandlich, Garth Cambray

**Affiliations:** 1 Faculty of Pharmacy, Pharmaceutical Chemistry Division, Rhodes University, Grahamstown 6140, South Africa; r.tandlich@ru.ac.za; 2 Makana Meadery, Reynolds St, Grahamstown 6139, South Africa; Meadery@gmail.com

**Keywords:** honey, ^1^H-NMR fingerprint, profiling, chemometrics, principal component analysis (PCA), partial least squares-discriminant analysis (PLS-DA), grouping

## Abstract

Honey is the natural sweet substance produced by honeybee from nectar or honeydew, exhibiting several nutritional and health benefits. It contains a complex mixture of compounds in different proportions, with sugars being the main component. The physicochemical characteristics of ten honeys were evaluated; represented by five, three, and two from South Africa, Slovakia, and Zambia, respectively. The range of values for the pH (3.75–4.38), electrical conductivity (99–659 µS/cm), and moisture content (14.2–17.7%) are within the recommended limits for quality honeys. ^1^H-NMR (Nuclear Magnetic Resonance) profiling of the honeys in *D_2_O* was determined, and the data were analysed by chemometrics. This method is fast, reproducible, and sample pre-treatment is not necessary. The ^1^H-NMR fingerprints of various chemical shift regions showed similarity or dissimilarity across geographical origins that are useful for identification, detection of adulteration, and quality control. The principal component analysis PCA and partial linear square discriminant analysis PLS-DA of the ^1^H-NMR profiles successively categorises the honeys into two chemically related groups. The R^2^ values are higher than the corresponding Q^2^ values for all samples, confirming the reliability of the model. Honeys in the same cluster contain similar metabolites and belong to the same botanic or floral origin.

## 1. Introduction

Honey is the natural sweet substance produced by honeybees (genera: *Apis* and *Meliponini*) after nectar or honeydew collection, transformation by combining with certain substances in the bee, and maturation; it is then deposited inside the beehive [1]. It is a complex mixture of more than seventy different compounds that are present in varying proportions, majorly natural sugars (fructose, glucose, maltose, and sucrose) [2]. The transformation process in honeybees involves regurgitation, evaporation, and enzymatic transformation of saccharides collected from nectar into honey [3].

Generally, the quality of honey and the chemical composition are linked to many factors, such as geographical, botanical or floral origin, climatic and seasonal, and botanical [4]. Other factors could be external, such as environmental factors, honey treatment methods by beekeepers, correct labeling of honey, storage conditions, and deliberate adulteration by producers [5]. For quality assurance purposes, these factors are important to honey producers, consumers, the food industry, and regulatory authorities. Africa is a diverse climatic region, which includes mangrove forests, tropical rain forests, savanna, and arid regions, with the honeys from these varied geographical and botanical origins presenting different chemical compositions that can be explored to categorise them into groups [4]. In general, honey metabolites include carbohydrates, protein, enzymes, minerals, amino acids and organic acids (e.g., formic, acetic), polyphenols, and carotenoids [6].The assessment of the origin of honey is important as part of the quality control requirements in line with the European Union (EU) regulation that the geographical and botanical origins of honey products must be printed on the label [4,7].

Several studies have been reported on the assessment of the physicochemical properties of honey in order to determine the quality, geographical, and botanical origins, and unravel possible adulteration associated with breeding, and the processing of natural and commercial products [8,9]. Natural honey is colourless to dark brown fluid; it can be viscous, partly, or entirely crystalline with varied organoleptic characteristics (e.g., taste, aroma) depending on age, length of storage, and botanical or floral origin [10]. Colour is a useful criterion for classifying unifloral honeys, and it often correlates with the flavour. Light-coloured honeys are mild-flavoured, while the dark ones have stronger flavour, and light-coloured honeys usually have higher prices than the dark ones [11]. The colour of honey can be determined using Pfund scale by measuring the level of absorption (in millimeters) of light using visible spectrophotometer at various wavelengths (i.e., 560 nm, Table 1) [12]. To the best knowledge of the authors, there is no previous report on the ^1^H-NMR profiling and chemometrics of South African and Zambian honeys. Also, little information is available on the spectroscopical assessment of Slovakian honeys that are being used in this study for comparison with African honeys.

Honey has many nutritional and health benefits [13,14,15,16,17,18,19,20,21], and it contains several useful nutritional substances, including monosaccharides and disaccharides (glucose and fructose, maltose, sucrose), group B vitamins and enzymes [3]. The rising commercial demand for honey worldwide has led to increase in the price of honey products, resulting in fraudulent adulteration of natural honey [22]. For instance, at least three times the quantity of Manuka honey that is produced in New Zealand is sold worldwide [22]. Therefore, beekeeping and commercial production of honey require more stringent quality control measures in order to deliver high-quality, safe products to the consumers.

Methods of honey adulteration include over feeding of bees with sucrose; addition of fructose or glucose, which may alter the fructose/glucose ratio (normal ratio in quality honey: 1–1.2); and feeding bees or addition of corn, invert sugar, and high fructose corn syrup (HFCS) with a resultant variation in the carbohydrate composition [23]. In recent times, adulterated honey has been on the increase, particularly with sugars such as glucose (30%), fructose (40%), maltose (8%), and sucrose (2%) [24]. The most frequent method of honey adulteration involves adding sucrose of greater than 1% of the dried mass, as well as rice syrup, because of its similar appearance and taste to natural honey [23,25]. Also, some honey adulterants are difficult to detect, such as brown rice syrup, BRS [26]. Hence, both the manufacturing process and the chemical composition of honey products are regulated in Europe by the EU Council directives [27]. Similarly, the Slovak Beekeepers Association (SZV) and Slovak Federation of Beekeepers (SVS) recommend and enforce stringent quality requirements for both locally produced and imported honeys [25]. SZV and SVS recommend that the hydroxymethylfurfural (HMF) content ≤ 15 mg/kg, water content ≤ 18%; they also specify the writing of a well-defined geographic origin on the label [28]. A high level of hydroxymethylfurfural (HMF) as a result of sugar metabolism in honey is an indication of heat damage and long storage time [29]. The objectives of the quality requirements in Slovakia were to increase national consumption of high-quality local honeys, reduce export of high-quality Slovak honey, lower importation of low-quality foreign honeys, and decrease both national purchase and consumption of low-quality honeys [28]. Several methods have been reported for the analysis of honey, aimed at assessing the quality and detection of adulteration. The conventional analytical techniques for quality assessment of honey from both unifloral and multifloral origins involve melissopalynological analysis in combination with chemometrics and the evaluation of organoleptic characteristics. These methods are time-consuming and operator dependent, and some category of adulterations (e.g., the addition of sugar concentrate to honey) are difficult to detect [30]. Other methods with specific advantages and limitations include mass spectrometry, high performance liquid chromatography (HPLC), and gas chromatography coupled with mass spectrometry (GC-MS) [31].

Nuclear magnetic resonance (NMR) spectroscopy in combination with chemometrics is useful in food industry for biomarker discovery and origin discrimination [32]. Application of NMR spectroscopy for the analysis of honey offers some advantages, including availability of a variety of information in a single measurement, which is relatively faster than most analytical methods in terms of run and processing time, reproducible and comparable data with high statistical confidence level, sample pre-treatment is not necessary, retrospective identification, and quantification of samples [33]. NMR spectroscopy is a useful approach for solving problems associated with the determination of the botanical and geographical origin of honey using pollen analysis, particularly when plants with similar pollen morphology are in abundance in the same area [33]. Also, ^1^H-NMR profiling can identify and quantify markers of different botanical origin; thus, it can be used to complement pollen analysis. Although other methods can differentiate chemical markers in honeys, they are usually cumbersome and require a combination of different methods [34,35]. When NMR spectroscopy is combined with chemometrics, overheating and the long storage condition of honey can be detected by monitoring specific markers, such as hydroxymethyl furfural (HMF) [26]. Also, ^1^H-NMR profiling can solve the problem associated with false positive results observed during quality control testing of some markers in honey that is adulterated with sugar when the Association of Official Agricultural Chemists, AOAC 998.12 C4 sugar detection test is used [36].

The current study is aimed at investigating the qualitative application of ^1^H-NMR fingerprints to honeys obtained from South Africa, Slovakia, and Zambia, to evaluate the clustering of the honeys using multivariate statistical tools. Thus, ^1^H-NMR profiling will assist in classifying the samples according to their botanical or geographical origins. A long term goal is to create a database of ^1^H-NMR fingerprints for honeys from Africa for the purpose of authenticating honey products, detecting adulteration, and for quality control.

## 2. Results and Discussion

### 2.1. Physicochemical Properties

#### 2.1.1. pH

The results of the pH (Table 2) showed values in the range of 3.75 ± 0.015 to 4.38 ± 0.025, which are within the recommended limit for a quality honey [1]. In general, the pH of quality honey ranges between 3.5 and 5.5 depending on the botanical origin, concentration of acids in the honey, pH of nectar and soil, ash content, and concentrations of minerals in the honey [37]. Apart from HD and HG with pH values of 4.01 ± 0.015 and 4.07 ± 0.01, respectively, other South African and Zambian honeys have higher pH values compared to those from Slovakia. High pH value increases the tendency for the formation of hydroxymethylfurfural, particularly during long storage time and when exposed to high temperature [38]. Although pH value does not directly relate to free acidity in honeys because of the presence of buffers and minerals, it is a parameter that contributes to the quality of honey and as a tool for evaluating the total acidity. When the pH value of honey is altered, it may indicate fermentation or adulteration [37].

#### 2.1.2. Electrical Conductivity (EC)

The honeys have electrical conductivity values in the range of 99 ± 5.03 to 659 ± 10.7 (µS/cm) that are below the recommended 800 µS/cm upper limit [1] (Table 2). There is a direct correlation between the acid content and EC of honeys. Additionally, EC has been a good criterion for botanical classification of botanical origin, and it is determined routinely [39]. The two Zambia honeys have high and similar EC values of 646 ± 5.0 and 659 ± 10.7 µS/cm for HI and HJ, respectively. These indicate that the two honeys are possibly obtained from the same botanical or floral origin.

#### 2.1.3. Moisture Content

The quality of honey is related to the moisture content. All the honeys (HA-HJ) have moisture contents less than the recommended upper limit of 20% [1,40] (Table 2). The lower the moisture content of a honey, the higher the shelf life and quality, since the degree of spoilage *via* fermentation by yeasts increases with the moisture content. HA (17.4 ± 1.95) from Slovakia, and HC (17.7 ± 0.330) and HE (17.4 ± 0.220) from South Africa have the highest moisture contents and may be more prone to fermentation and granulation than the other honeys during storage [41]. Generally, high moisture content becomes a great challenge for preserving the quality of honey when the storage temperature and relative humidity are high [42].

### 2.2. ^1^H-NMR Fingerprinting

Nuclear magnetic resonance (NMR) spectroscopy has been used to identify organic compounds in food samples, particularly liquids such as wines, fruit juices, and honeys [36,43]. For example, some Italian honeys were classified using NMR spectroscopy in combination with multivariate statistical tools [44,45], and the principal component analysis (PCA) of the NMR data has been used to determine the identity card of saccharides from floral source [46,47]. The chemical composition of natural honey depends principally on the floral origin and geographical source; thus, the chromatographic and spectroscopic fingerprints are not expected to be the same for the honeys under current investigation, since they were obtained from different geographical and floral origins.

**Figure 1:**
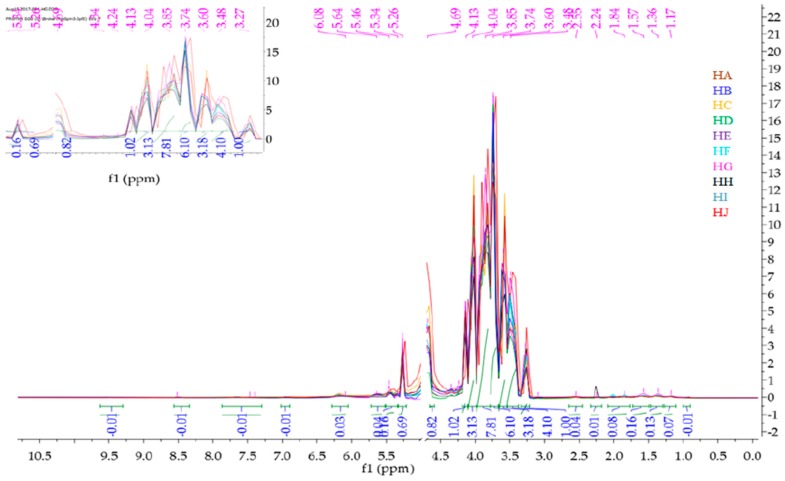
Superimposed and binned full ^1^H-NMR spectra of honeys HA-HJ (10.5–0 ppm) in *D_2_O*, using Mnova 12 NMR software.

**Figure 2:**
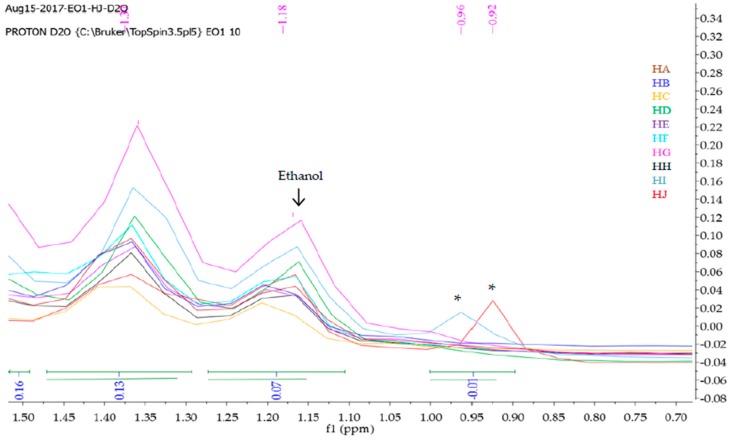
Fingerprints of superimposed and binned ^1^H-NMR spectra in *D_2_O* at region 1.50–0.75 ppm, using Mnova 12 NMR software. * Variable Importance in Projection (VIP) chemical markers resonating at 0.92 and 0.96 ppm.

**Figure 3:**
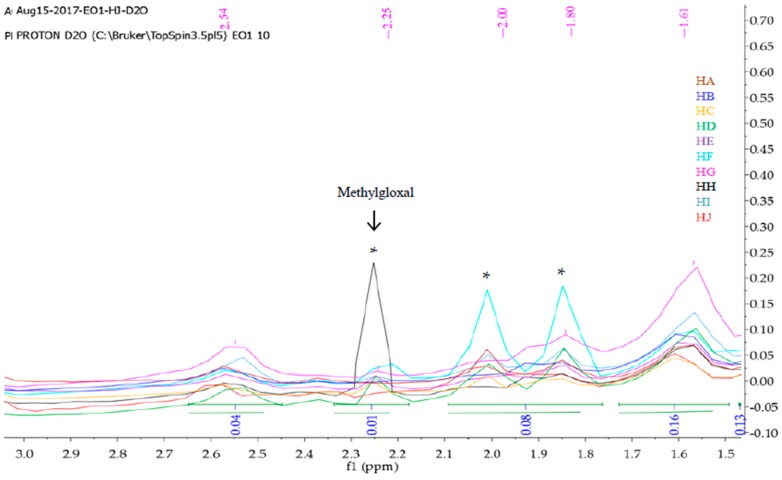
Fingerprints of superimposed and binned ^1^H-NMR spectra in *D_2_O* at region 3.00–1.50 ppm, using Mnova 12 NMR software. * VIP chemical markers resonating at 1.80, 2.00, and 2.17 ppm.

**Figure 4:**
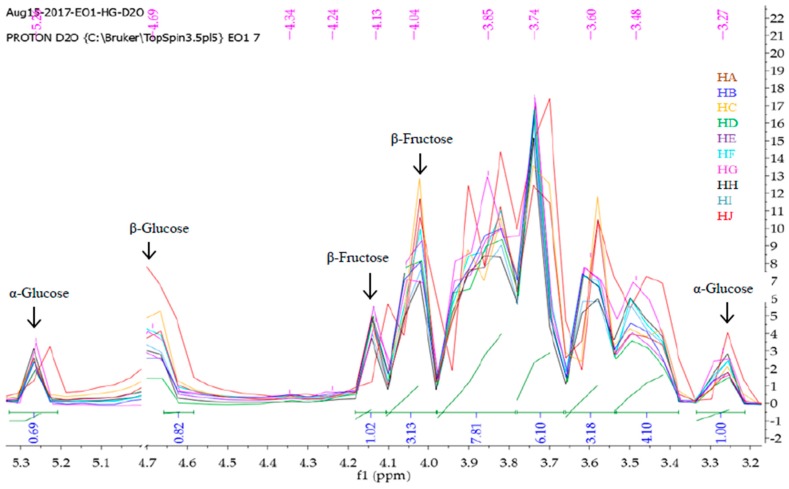
Fingerprints of superimposed and binned ^1^H-NMR spectra in *D_2_O* at region 5.30–3.20 ppm, using Mnova 12 NMR software.

**Figure 5:**
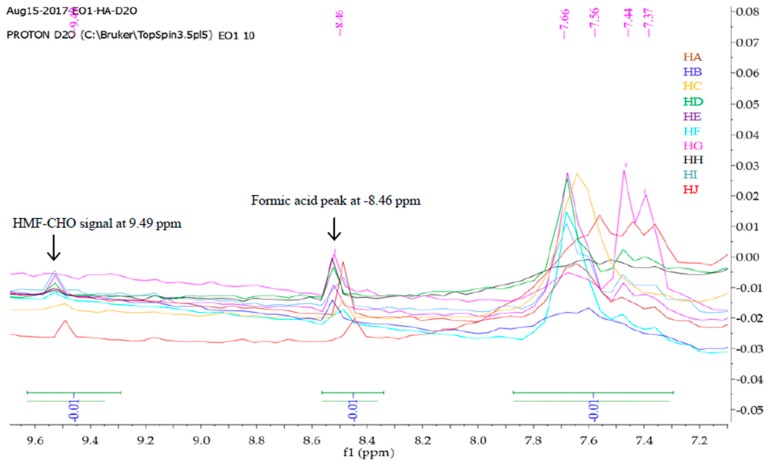
Fingerprints of superimposed and binned ^1^H-NMR spectra in *D_2_O* at region 9.60–7.20 ppm, using Mnova 12 NMR software [49].

Mnova 12 NMR software was used for the ^1^H-NMR fingerprinting. The zone between 4.70 and 5.00 ppm was excluded from the superimposed and binned spectra of HA-HJ in order to get rid of the residual water peak [26]. Then, the stacked ^1^H-NMR spectra signals between *δ* values 10.5 and 0 ppm (Figure 1) were segmented into various regions (9.60–7.20, 5.30–3.20, 3.0-1.50, and 1.50–0.75) to obtain characteristic fingerprints for each of the honeys to identify and classify the samples on the basis of similarity or dissimilarity of their chemical composition (Figure 2, Figure 3, Figure 4 and Figure 5). The full superimposed and binned spectra (Figure 1) at *δ* value range of 10.5–0.00 ppm revealed similar fingerprints for all the honeys, showing possible similarities in the major chemical composition. However, the weak signal regions (0.75–1.50, 3.00–1.50, and 9.60–7.20 ppm) of the expanded spectra showed characteristic peaks for each honey, which can be used to categorise them into clusters. The fingerprints of all the honeys at region 1.50–0.75 ppm are similar to the exception of the two Zambia honeys (HI and HJ) with an extra signal at 0.93 ppm (Figure 2), indicating the same floral origin for HI and HJ. It was also observed that each of the honeys has a diagnostic fingerprint at region 3.00–1.50 ppm with the exception of the South African (HH) and a Zambian (HI) honeys that have similar pattern (Figure 3), indicating similar chemical constituents in this region of the spectra origin. Region 9.60–7.20 ppm (Figure 5) represents the downfield region, showing HA, HC-HF, and HI sharing similar fingerprints, while each of the remaining honeys (HB, HG, HH, and HJ) has a unique fingerprint in this region of the spectra. This shows that apart from HB and HH, all honeys obtained from South Africa, HA from Slovakia, and HI from Zambia contain similar chemical constituents in this region. However, irrespective of the geographical and botanical origin, all the honeys contain two chemical markers with *δ* values of 8.46 ppm (formic acid) and 9.49 ppm (HMF) [48]. The carbonyl signal CHO resonating at 9.49 ppm has a low integral value 0.02 compared to the major signals, as typified by α-Glucose signal resonating at 3.25 ppm with an integral value of 1. It shows that all the honeys contain hydroxymethyl furfural (HMF), which is a quality control marker in honeys. Generally, the level of HMF in honey increases during prolonged storage and with overheating of honey during processing. Other signals observed in the weak fingerprint regions include ethanol (1.18 ppm, CH_3_), methylgloxal (2.25 ppm, CH_3_), and other aryl signals at 7.41 and 7.66 ppm (Figure 2, Figure 4 and Figure 5) [3,49]. The ^1^H-NMR signals at the most intense region (*δ* = 5.30–3.20 ppm), particularly between 4.20 and 3.20 ppm, represent the major monosaccharides (e.g., d-glucose and d-Fructose) and disaccharides (e.g., maltose and sucrose) in honey (Figure 4), whereas the 5.30–4.50 ppm portion of this region contains mainly anomeric and glycosidic signals, and all the honeys have similar ^1^H-NMR fingerprints in this region. Thus, this region cannot be used to categorise or distinguish the honeys as corroborated by the assignment of the ^1^H-NMR signals for the major monosaccharides in honey [46].

### 2.3. Data Validation and Transformation

Model selection, validation, transformation, and quality control of the raw data were carried out using MetaboloAnalyst 3.0 (Parasitology Building, 21111 Lakeshore Road Ste. McGill University, Anne de Bellevue, QC, Canada; http://www.metaboanalyst.ca), Simca 15 (Umetrics, Tvistevägen 48 SE-907 36, Umeå, Sweden) and the Unscrambler^®^ X 10.5 (CAMO Software AS., Oslo Science Park, Gaustadalléen 21, Oslo, Norway) software. Variation in the intrinsic pH of honeys may cause the chemical shifts of the chemical constituents or metabolites to vary, and it may require the addition of buffers to the deuterated solvents for the NMR experiments. The preliminary investigation of the pH values of the honeys showed a narrow pH range with a value of 0.63 (calculated from the difference between 3.75 ± 0.015 and 4.38 ± 0.025); thus, the addition of buffers to *D_2_O* was considered unnecessary. The ^1^H-NMR data points were reduced from 65534 data points to 271 continuous bins of equal width of 0.04 ppm for the chemical shift range of 10.5–0.00 ppm, using Mnova 12 NMR software after peak peaking and integration of the spectra. This procedure was carried out to reduce dimensionality in chemical shifts among the honeys, which may arise from small variations in pH, ionic environment, and other factors that may affect the chemical environment and the results of the principal component analysis (PCA) and principal least square discriminant (PLS-DA) methods.

**Figure 6:**
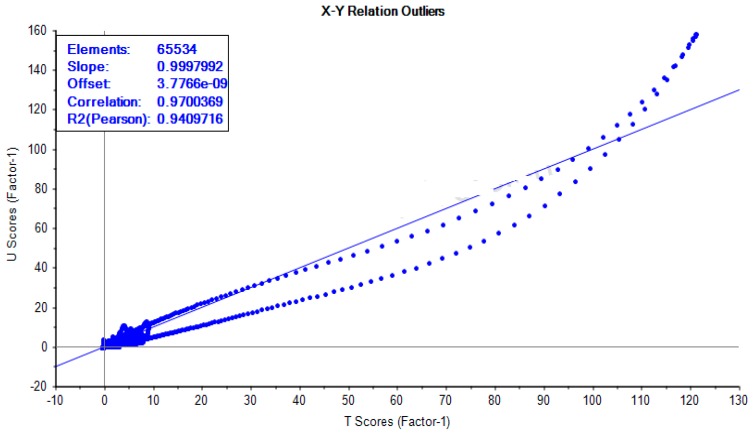
Outliers plot of U-scores vs. T-scores of the H-NMR spectra of honeys (HA-HJ) using Unscrambler^®^ X 10.5 software.

**Figure 7:**
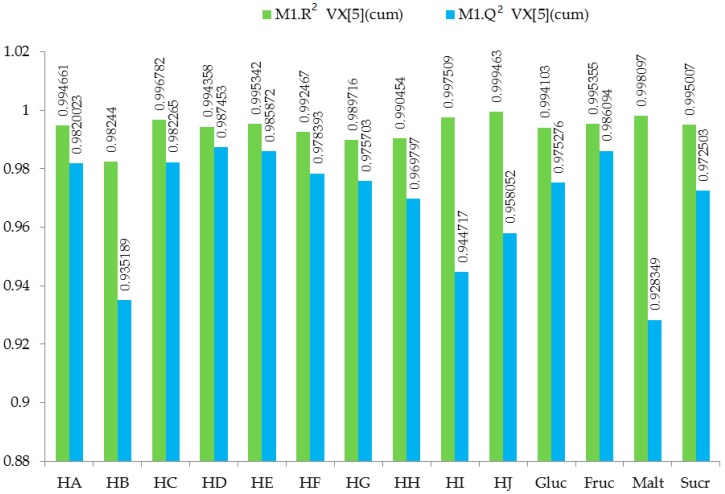
Cumulative cross-validation values for R^2^ and Q^2^ for HA-HG and the reference sugars, using Simca 15 software.

**Figure 8:**
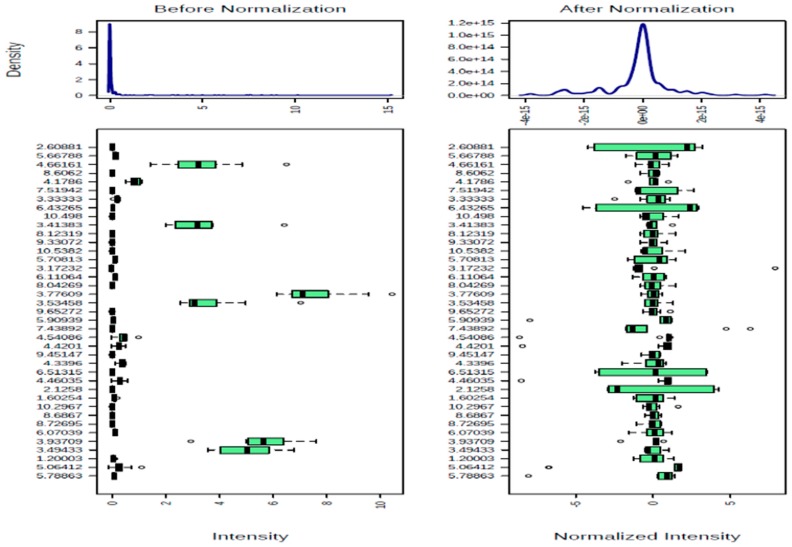
Log 2 transformed normalisation of HA-HJ using Pareto scaling for MetaboloAnalyst 3.0 software.

All the OH peaks of the sugars of the ^1^H-NMR spectra of the honeys have been exchanged for *D_2_O*, and they appeared as broad peaks at 4.790 ppm. Hence, region 5.00–4.79 ppm was excluded from the spectra to remove the effect of variation of the suppression of the water resonance and any cross-relaxation effect of the OH peaks of the honeys that has been exchanged for *D_2_O*. The Unscrambler^®^ X 10.5 software was used to determine the outliers, representing data points that arise from a different model and that are different from the rest of the data points, showing correlation coefficient of 0.97004 and R^2^ value of 0.94097, indicating good linearity of the ^1^H-NMR data for HA-HJ (Figure 6). The results of cross-validation of five cycles using with Simca 15 software showed the R^2^ values to be higher than the corresponding Q^2^ values for HA-HJ and the reference sugars (Figure 7), confirming the reliability of the model. Similarly, cross-validated R^2^Y and Q^2^ coefficients values of 0.989 and 0.477, respectively, and PLS-DA model validation based on separation distance of *p* value of 0.797 were obtained using permutation tests for MetaboloAnalyst 3.0 software. The ^1^H-NMR spectra of d-glucose, d-fructose, maltose, and sucrose in *D_2_O* were similarly treated using Mnova 12 NMR, and were used as the reference values for the main metabolites of the honeys. Figure 8 showed the effect of quantile normalisation of the data using Log 2 transformation and Pareto scaling to make the metabolites concentration data reasonably normal and more comparable [50].

### 2.4. ^1^H-NMR Metabolomics Using Multivariate Analysis

^1^H-NMR spectroscopy in combination with chemometrics is a simple, fast, and reproducible method for profiling the metabolites in honey, and it only requires about 30 minutes for spectra collection, processing, and analysis of data. Recently, ^1^H-NMR profiling has become a fast and global method for authenticity screening for honeys [51]. The botanical origin of honey can be determined by selected signals using Principal Component Analysis (PCA) and projection to latent structures by Partial Least Square-Discriminant analysis (PLS-DA) [51]. In current work, the PCA and PLS-DA were performed using MetaboloAnalyst 3.0 software to visualise and evaluate the ^1^H-NMR data, report correlation, and categorise the samples.

**Figure 9:**
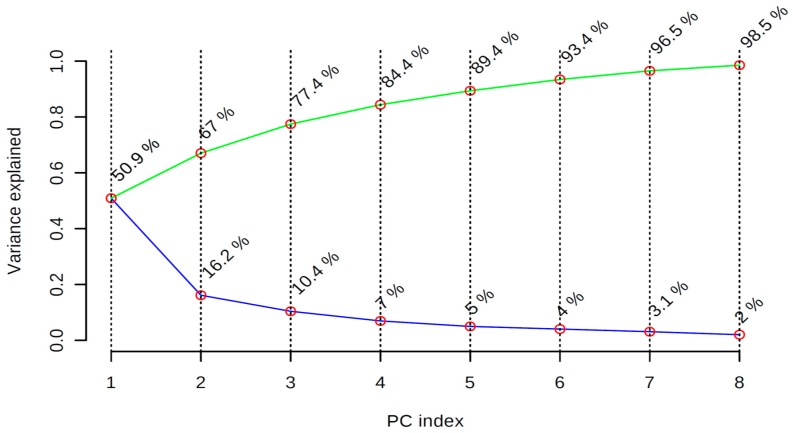
Scree plot showing the variance explained by PC1-PC8 using MetaboloAnalyst 3.0. Green line: accumulated variance. Blue line: variance by individual PC.

Generally, the two most commonly used multivariate statistical tools are PCA and PLS-DA, and the results are often presented as loadings and scores. Score plots are two or three dimensional plot of the projection of the raw ^1^H-NMR data into a new coordinate system of principal components (e.g., PC1 and PC2 axes) to explain the maximal variance (PCA) or co-variance (PLS-DA) of the data. The score plot represents the map of the honey, showing the degree of closeness in the square plot as an indication of the similarity or dissimilarity of the binned data for the ^1^H-NMR intensity measured at 10.5-0.00 ppm range, whereas loading plots often show the composition of the variable or compounds responsible for the discriminating patterns. The PCA scree plot of the variance explained by PC1-PC8 showed that the principal component PC1 contributed 50.9% to the total variance observed. The other significant components are given by PC2-PC5 with percentage contributions of 16.2%, 10.4%, 7%, and 5%, respectively. The result further showed that the first eight components accounted for 98.5% of the variance of the samples data matrixes (Figure 9).

**Figure 10:**
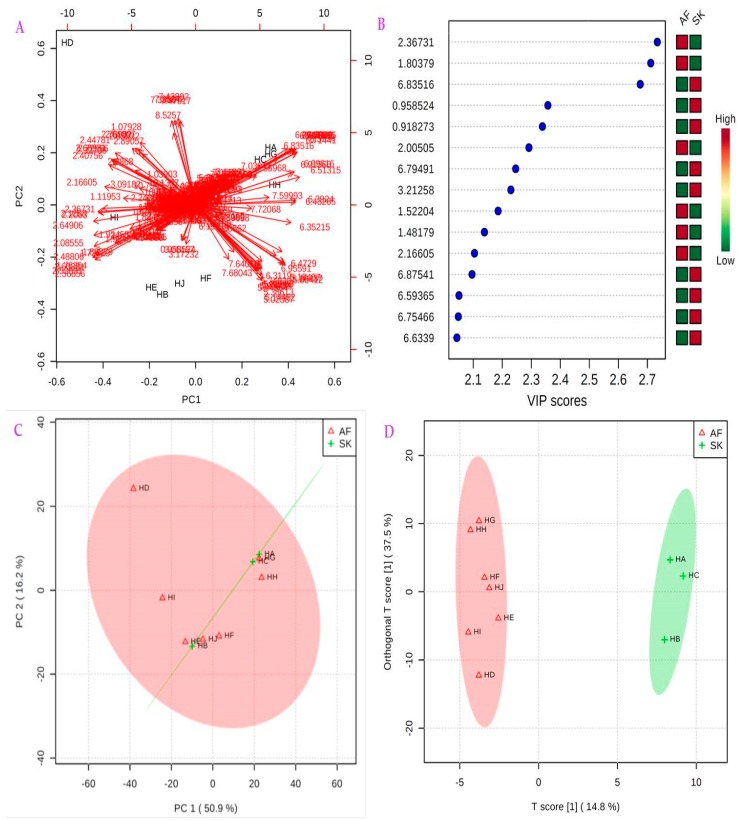
Screen reports for HA-HJ for MetaboloAnalyst 3.0. (**A**) PCA biplot of PC1 vs. PC2; (**B**) Important chemical shifts of major compounds identified by PLS-DA with the coloured boxes indicating the relative concentrations of the corresponding metabolites in African (AF) and Slovakian (SK) honeys; (**C**) PCA score plot of PC1 vs. PC2, showing the explained variances in brackets; (**D**) OPLS-DA scores plot of all metabolite in HA-HJ.

PCA is an unsupervised multivariate analytical method useful for summarising or reducing dataset into smaller number of independent variables. It is the weighted average of the original variables, which aims to find the directions that best explain the variance among the variables (X) without making reference to the class labels (Y). PCA extracts information and can identify trends or characteristics within the data; it removes noise during processing of the variables to explain structured variance and reduces dimensionality by compressing the data. It is a superior tool for exploratory data analysis and it is one of the bases for discriminating, classifying, and identifying samples during multivariate analysis. Variable reduction is also possible with PCA using process monitoring to get a virtual analysis of variance [52,53]. Besides scores and loadings, PCA can also be presented as a biplot, a two dimensional scattered plot that offers information derivable from both loadings and scores plots in a single chart. The PCA biplot of the two main principal components PC1 and PC2 (Figure 10A) for MetaboloAnalyst 3.0 software showed two Slovak honeys (HA and HC) and two South African honeys (HG and HH) that were to be categorised in the same cluster. Apart from HI from Zambia and HD from South Africa belonging to distinct groups, the remaining honeys from Slovakia (HB), South Africa (HE and HF), and Zambia (HJ) were discriminated into the same group by certain chemical markers. The results of the PCA score plot confirm these groupings as shown in the explained variance in the brackets (Figure 10C).

The PLS-DA of the binned and normalised ^1^H-NMR dataset of HA-HJ was carried out to extract linear combination of the original variables using regression techniques in order to predict the class membership of the variables. PLS-DA is a supervised method that can be used for classification and feature selection of the variables, using cross-validation, to select an optimal number of components for classification [54]. The results of variable importance in projection (VIP) of the relevant chemical shifts of the discriminating compounds as identified by PLS-DA with the colored boxes are presented in Figure 10B, showing the relative concentrations of the corresponding metabolites in African (AF) and Slovakian (SK) honeys. VIP is a weighted sum of squares of the PLS loadings with reference to the amount of explained Y-variation in each dimension, and VIP scores are usually calculated for each component. Figure 10B showed certain compounds with the highest VIP values at various concentrations that can be used to discriminate the honeys into clusters or groups. The results also revealed that Slovakian honeys contain two chemical markers with high VIP values and high concentrations, namely methylgloxal resonating at 2.25 ppm and another compound resonating at 1.80 ppm (Figure 3 and Figure 10B). Other markers in honeys from Slovakia with relatively high concentrations and VIP values include compounds resonating at 2.17, 2.00, 1.52, and 1.48 ppm (Figure 2, Figure 3 and Figure 10B). Similarly, the African honeys contain nine chemical markers with relatively high concentrations compared to the Slovakian honeys, including compounds resonating at 6.84, 0.96, 0.92, 6.79, 6.87, 6.59, 6.75, and 6.63 ppm, and they are arranged in the order of decreasing VIP values. These results further showed that compounds of relevance in discriminating between honeys from South Africa, Zambia, and Slovakia are present in relatively very low concentrations compared to the major components of honeys, particularly the four reference sugars (i.e., glucose, fructose, maltose, and sucrose). Hence, further studies are necessary to identify and quantify the phytochemical constituents of these honeys that are present both in high and low concentrations using titrimetric, chemical, and spectroscopical methods.

We also performed the Orthogonal-Orthogonal Projections to Latent Structures Discriminant Analysis (OPLS-DA). This is a supervised modeling method and a powerful tool for dimensions reduction and identification of spectral features for cluster separation, and it makes models less complex and more meaningful than PLS-DA. OPLS-DA may be used instead of PLS-DA, arising from its ability to discriminate between variations in the normalised ^1^H-NMR dataset of HA-HJ that are important for predicting clustering and variations irrelevant to predicting groupings. The results of the OPLS-DA score plot (Figure 10A,C) successfully grouped the honeys from Slovakia and Africa into clusters, corroborating the results of the PCA and biplot scores.

**Figure 11:**
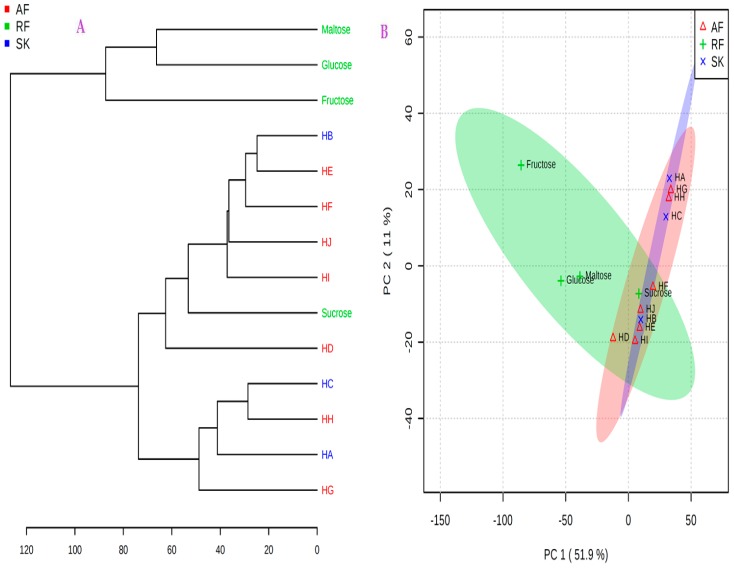
Screen reports for HA-HJ & reference sugars using MetaboloAnalyst 3.0 software. (**A**) Dendrogram clustering, showing the distance measured for African honeys (AF), Slovak honeys (SK), and the reference sugars (RF), using Euclidean option and complete clustering algorithm; (**B**) PLS-DA score plot of PC1 vs. PC2, showing the explained variances in brackets.

Further analyses were carried out with MetaboloAnalyst 3.0 software to examine the correlation between the honeys and the major sugars (i.e., glucose, fructose, maltose, and sucrose) in the honeys, and to determine the discrimination patterns among the HA-HJ using the these sugars as the reference standards (Figure 11A,B). Hierarchical cluster analysis was performed using average clustering algorithm, centroids option was used for the observations, and the distance was measured using Euclidean. The dendrogram (Figure 11A) and the PLS-DA score plot (Figure 11B) both showed two major clusters, including the first cluster comprising two Slovakia honeys (HA & HC) and two South Africa honeys (HG & HH). The remaining honeys were discriminated into the second cluster along with sucrose. The latter cluster shows that the Slovakian honey HB, three South African honeys (HD-HF), and the two Zambian honeys (HI & HJ) contain relatively higher levels of sucrose compared to honeys in the first cluster. If these values, along with other sugars and chemical markers in honey, are within the recommended limits, it suggests that honeys in the second group are of better quality. However, if the levels of sucrose in the second cluster are higher than the recommended limit, possible adulteration may be suspected either by direct addition of rice syrup, invert sugar, or corn syrup, or by indirect adulteration by feeding bees with sucrose by the beekeepers.

## 3. Methods

### 3.1. Samples and Chemicals

A total of ten honey samples were used for the study, representing Slovakia (3: HA-HC), South Africa (5: HD-HH), and Zambia (2: HI & HJ). The South African and Slovak honeys were purchased from the supermarket, while Dr. Cambray Garth of Makana Meadery, Grahamstown, South Africa obtained the Zambian honeys directly from the beekeepers. All chemicals and standards (fructose, glucose, maltose, and sucrose) that were used to qualify the major compounds present in the honey samples were of high analytical purity. Deuterium oxide (99.9 atom % D) was purchased from Sigma-Aldrich, Johannesburg, South Africa.

### 3.2. pH

The method described by de Moraes and Teixeira [55] was used. Each honey (6 g) was suspended in distilled water (45 mL) and homogenised on a shaker (Already Enterprises Inc., Taiwan) at 200 rpm at room temperature to obtain a 13.3% (*w*/*v*) solution. Thereafter, the pH meter was calibrated using buffer solutions with pH values of 7.0 (Minema^®^ B7070) and 4.0 (Minema^®^ B6760), and the pH of the honey solutions were measured in triplicates [55].

### 3.3. Electrical Conductivity

The electrical conductivity was measured using a portable Electrical conductivity (EC) meter. A 13.3% (*w*/*v*) solution of each honey was prepared by dispersing 6 g of honey in distilled water (45 mL) and homogenised on a shaker (Already Enterprises Inc., Taiwan) at 200 rpm at room temperature. The EC meter was calibrated using a conductivity standard 1415 μS/cm buffer (Minema^®^ 1413 uSIEMENS/cm), and the EC of the honey solutions were measured in triplicates [56,57].

### 3.4. Moisture Content

The procedure described by Mairaj et al. [58] was adapted and used to determine the moisture contents (%) of the honeys in current studies. Crucibles were placed in an oven at 55 °C for 30 min and cooled to room temperature in a desiccator. About 2 g of each honey was weighed into the crucible in triplicate, and the actual weight of the honey was calculated. Then, the honeys were heated at 100 °C for 18 h in an oven and cooled in a desiccator to the room temperature, and the weight of the residual honey was determined using (1) equation below.

(1) $$\mathrm{Moisture}\;\mathrm{content}\;(\%)=\frac{\mathrm{Weight}\;\mathrm{of}\;\mathrm{fresh}\;\mathrm{honey}-\mathrm{weight}\;\mathrm{of}\;\mathrm{dry}\;\mathrm{honey}}{\mathrm{Weight}\;\mathrm{of}\;\mathrm{fresh}\;\mathrm{honey}}=\frac{w2-w3}{w2-w1}$$

where: w1 = weight of crucible, w2 = weight of crucible & fresh honey, w3 = weight of crucible & dry honey.

### 3.5. Sample Preparation and NMR Data Pre-Treatment

The honey samples were homogenised at 200 rpm for five minutes on a shaker (Already Enterprises Inc., Taiwan) at room temperature before sampling to ensure a homogenous mix, and 50 µL of each sample was dissolved in 350 µL of Deuterium oxide (*D_2_O*, 99.9 atom % D, Aldrich) and then transferred into NMR tube with 5 mm outer diameter. The ^1^H-NMR spectra of the honeys in *D_2_O* were acquired at 300 K on Bruker Avance^TM^ III HD 400 MHz spectrometer, TopSpin 3.5 pls (Bruker BioSpin, Rheinstetten, Germany) with a SampleXPress autosampler (Bruker BioSpin, Rheinstetten, Germany) using a PULprog Zg30 at 300 K. The spectra were measured at the frequency of 400.13 Hz by taking 16 scans with 2 prior dummy scans, spectra width (SW) of 20.0254 ppm (or 8016.821 Hz), receiver gain of 32, time, and frequency domain of 32767 and 262144 points, respectively, as well as pulse width of 10.5 μs, relaxation delay of 1.0 s, and acquisition time of 4.096 s. The NMR chemical shifts are reported in ppm, and the spectra were calibrated with residual water signal (*δ*: 4.790 ppm) before aligning the spectra to compensate for the intrinsic acidity of different honey sample.

The ^1^H-NMR spectra of the four references (d-glucose, d-Fructose, maltose, and sucrose in *D_2_O*) were recorded and used to determine the response factor of the honeys. Zones without useful information beyond 10.5 ppm and before 0 ppm were removed. The proton signals were standardised using automatic positive peak picking and interactive default options, and both known solvents and impurities were auto-classified (hidden). The ^1^H-NMR spectrum of each of the honey samples HA-HJ and the four reference sugars was processed separately using Mnova 12 (Mestrelab Research S.L., Santiago de Compostela, Spain). Each spectrum was zero fill to a high value of 512K using Toeplitz method with base point and coefficient values of 32743 and 24, respectively. Then, the signals were normalised with the largest peak scale set at 100 in order to make more meaningful comparisons between the samples and smoothed along f1 using auto-directed Whittaker smoother with a smooth factor of 145. This was followed by a series of processing, including Fourier transformation along t1 using Swap Halved (quadrature) approach, automatic phase correction by metabolomics algorithm, and baseline correction using full auto-Bernstein polynomials of degree 5. Positive peak peaking option was used, involving a noise factor of 250, and a maximum number 1000 peaks with a parabolic interpolation and interactive option. The ^1^H-NMR signals were integrated manually for each spectrum and normalised to make variables more comparable. Thereafter, the HA-HJ spectra were superimposed, aligned, and binned to remove the problem of chemical shift variations with width of each chemical shift region of 10.50–0.00 ppm range set at 0.04 ppm. This approach was employed to solve complications arising from the dependence of the chemical shifts on temperature, pH, and ionic strength of the samples that can influence the electronic environment and the fingerprinting datasets of the metabolites acquired from NMR spectrometers [59]. Then, the stacked and binned spectra in the range of 0–10.5 ppm were segmented into four regions (i.e., 0.75–1.50, 3.00–1.50, 5.30–3.20, and 9.60–7.20 ppm), zoomed, and compared with literature reports. The responses of the four sugars were used as the standards during statistical processing of the data. The OH signals of the sugars in HA-HJ and reference sugars at 4.70–5.00 ppm have been completely exchanged with *D_2_O*, and were excluded from the analysis to get rid of the residual water peak [26,60,61,62].

### 3.6. Multivariate Data Analysis

The Unscrambler^®^ X version 10.5 (CAMO Software AS., Oslo, Norway), Simca 15 (Umetrics, Umeå, Sweden), MetaboloAnalyst 3.0, and Mnova 12 NMR (Mestrelab Research S.L., Santiago de Compostela, Spain) software were used for all chemometric treatments, including visualisation, processing, analysis, and reporting. Raw ^1^H-NMR spectra were superimposed and binned using Mnova 12, saved as ASCII text files (*.txt), then imported to Microsoft Excel and saved as Excel 97-2003 workbook (*.xls) or CSV comma (*.csv) files. Comma delimited or separator files of CVS and Excel 97-2003 workbook files were imported to Simca 15 and other software for multivariate analysis.

Typical general procedures that were carried out on the datasets for each part of the software before multivariate analysis of the binned and integrated ^1^H-NMR data are explained for MetaboloAnalyst 3.0 software. These include data check for missing values, data filtering using mean intensity value, quantile normalization (for HA-HJ dataset) or normalisation by a pooled sample from the reference sugar group (for HA-HJ & reference sugars dataset) using Log 2 transformation and Pareto scaling in both cases, and cross-validation of the normalised data using LOOCV method with the performance measure set at Q^2^. Cross-validation procedure was further cross-checked using Simca 15 software for HA-HJ and the reference sugars in order to ascertain the reliability of the model.

## 4. Conclusions

The physicochemical characteristics and ^1^H-NMR profiling of ten selected honeys from South Africa (five), Slovakia (three), and Zambia (two) were examined, and the spectroscopy data were analysed using Chemometric statistical tools. The values for the pH (3.75–4.38), electrical conductivity (99–659 µS/cm), and moisture contents (14.2–17.7%) are within the recommended limits. The ^1^H-NMR profiling of various chemical shift (*δ*) regions clearly showed that each honey has a diagnostic fingerprint with specific chemical markers that can be used for identification, detection of adulteration, and, ultimately, for quality control purpose. Multivariate analysis of the normalised and binned data using principal component analysis (PCA) and partial linear square discriminant analysis (PLS-DA) showed linear correlation and successively categorised the honeys into two distinct clusters. Two of the Slovak honeys (HA and HC) contain similar metabolites as HG and HH from South Africa. These four honeys exhibited lower levels of *sucrose* relative to the remaining honeys, indicating better quality provided if the sucrose is within the recommended limit. The second group includes the two Zambian honeys (HI and HJ), HB from Slovakia, and three South Africa honeys (HD, HE, and HF) with closer correlation in the levels of four sugars, particularly sucrose, glucose, and maltose. If the concentrations of the chemical constituents of the latter group are within the acceptable limits, it may suggest that these honeys are of better quality than the first group. In summary, the honeys have been categorised into two chemically related groups using ^1^H-NMR spectroscopy in combination with chemometrics, indicating possibly two different botanical or floral origins in the selected honeys from South Africa, Slovakia and Zambia.

## Figures and Tables

**Table 1:** Pfund scale for determination of honey color.

Colour	Pfund Sacle (mm)	Colour Range
Water white	1 to 8	0.030 or less
Extra white	More than 8–17	More than 0.030–0.060
White	More than 17–34	More than 0.060–0.120
Extra light amber	More than 34–50	More than 0.120–0.188
Light amber	More than 50–85	More than 0.188–0.440
Amber	More than 85–114	More than 0.440–0.945
Dark amber	More than 114	More than 0.945

**Table 2:** Electrical conductivity (µS/cm), moisture content (%), and pH of honeys HA-HJ.

Honey	Geographical	pH	Moisture Content	Electrical Conductivity
Code	Origin		(%)	×10^2^ (µS/cm)
HA	Slovakia	4.06 ± 0.006	17.4 ± 1.95	4.29 ± 0.0503
HB	Slovakia	4.01 ± 0.015	14.6 ± 0.116	1.79 ± 0.0404
HC	Slovakia	3.75 ± 0.015	17.7 ± 0.330	0.99 ± 0.0503
HD	South Africa	4.01 ± 0.015	15.0 ± 0.180	4.66 ± 0.0252
HE	South Africa	4.16 ± 0.015	17.4 ± 0.220	4.12 ± 0.111
HF	South Africa	4.27 ± 0.012	14.4 ± 0.19	4.19 ± 0.105
HG	South Africa	4.07 ± 0.01	14.2 ± 0.060	2.17 ± 0.105
HH	South Africa	4.38 ± 0.025	17.4 ± 0.120	1.78 ± 0.0436
HI	Zambia	4.29 ± 0.01	15.0 ± 0.350	6.46 ± 0.050
HJ	Zambia	4.29 ± 0.012	16.8 ± 1.15	6.59 ± 0.107

Values are expressed as mean ± stand deviation in triplicates with *p* < 0.05.
